# Advances in the Application of Phytogenic Extracts as Antioxidants and Their Potential Mechanisms in Ruminants

**DOI:** 10.3390/antiox12040879

**Published:** 2023-04-04

**Authors:** Minyu Piao, Yan Tu, Naifeng Zhang, Qiyu Diao, Yanliang Bi

**Affiliations:** Key Laboratory of Feed Biotechnology of the Ministry of Agriculture and Rural Affairs, Institute of Feed Research, Chinese Academy of Agricultural Sciences, Beijing 100081, China

**Keywords:** oxidative stress, reactive oxygen species, plant extracts, antioxidant, ruminants

## Abstract

Under current breeding conditions, multiple stressors are important challenges facing animal husbandry in achieving animal wellbeing. For many years, the use of antibiotics has been a social concern in the livestock industry. With the implementation of the non-antibiotics policy, there is an urgent need to find relevant technologies and products to replace antibiotics and to solve the problem of disease prevention during animal growth. Phytogenic extracts have the unique advantages of being natural and extensive sources, having a low residue, and being pollution-free and renewable. They can relieve the various stresses, including oxidative stress, on animals and even control their inflammation by regulating the signaling pathways of proinflammatory cytokines, improving animal immunity, and improving the structure of microorganisms in the gastrointestinal tract, thereby becoming the priority choice for improving animal health. In this study, we reviewed the types of antioxidants commonly used in the livestock industry and their applicable effects on ruminants, as well as the recent research progress on their potential mechanisms of action. This review may provide a reference for further research and for the application of other phytogenic extracts and the elucidation of their precise mechanisms of action.

## 1. Introduction

Oxidative stress (OS) is defined as a state of imbalance between oxidation and antioxidation in the body, with oxidation tending to prevail, leading to inflammatory neutrophil infiltration, increased protease secretion, and a large production of oxidative intermediates. Oxidative stress is a negative effect induced by free radicals. Free radicals are intermediate products or byproducts of cell metabolism, and they include reactive oxygen species (ROS) and reactive nitrogen species (RNS), which are mainly generated in the process of adenosine triphosphate synthesis by mitochondria [1]. Under normal physiological conditions, ROS and RNS are important components involved in various cellular biological processes, such as signal transduction, defense against viral infections, and redox regulation [2]. However, an excessive accumulation of free radicals causes OS, affecting the structure and physiological functions of cells, which can contribute to poor health in livestock. Oxidative stress has long been an active research field in ruminant medicine, as it is implicated in various metabolic diseases, including sepsis, acidosis, enteritis, ketosis, mastitis, pneumonia, respiratory, and joint disease [3]. The internal and external environment stressors, dietary problems (excessive unsaturated fatty acids, trace elements and vitamins shortage, and pesticides and herbicides residues), and the changes in the animal’s physiology and psychology can cause the accumulation of peroxides in their body, leading to oxidative damage [4]. During the metabolism of organic substances, such as carbohydrates, fats, and proteins, the oxygen molecules are generally reduced to water through four steps of single-electron reduction under the action of the mitochondrial respiratory chain, during which it is first necessary to form superoxide anions (O_2_^•−^), which then combine with hydrogen ions (H^+^) to finally generate water. However, some superoxide anions fail to be completely reduced in this reaction, leading to the generation of superoxide. The superoxide can be converted to hydrogen peroxide (H_2_O_2_) and then water or toxic hydroxyl. The accumulation of ROS is accompanied by the production of RNS, such as toxic peroxynitrite, which is formed by the reaction between ROS and nitric oxide (NO). More radicals are generated under conditions of inefficient electron transfer, and nitrative and oxidative damage of mitochondrial proteins aggravates radical production, leading to calpain activation, excitotoxicity, and the promotion of proapoptotic cascade activation [5].

Exogenous antioxidants are usually supplemented in livestock diets to relieve the OS of animals and to prevent the deterioration of easily oxidized components in the diet. However, due to the fact that it is difficult to precisely estimate the degree of OS in animals, some excessive synthetic antioxidants are usually added to their diets and fed to them, leading to excess additive residues in animals and, consequently, in their products. Phytogenic extracts, referred to as plant-derived compounds or plant extracts, are bioactive substances with unique functional groups. These extracts are known to be sustainable additives; are used to enhance animal performance and health [6]; and possess antioxidant, antimicrobial, anti-inflammatory, and immunostimulant properties [7]. Plant extracts commonly include polysaccharides (such as achyranthes bidentata polysaccharides), polyphenol compounds (such as resveratrol and proanthocyanidins), alkaloids (such as sinapine), and saponins (such as steroid saponin). Based on the different chemical structures of various plant extracts, the animal responses and the modes of action are different [8]. In this review, we summarize the recent findings on the application of phytogenic extracts in ruminants, as well as their various mechanisms of action.

## 2. Oxidative Stress, Inflammation, and Defense Strategies

In livestock production, animals are commonly unavoidably exposed to various stressors, including external factors, such as climate, transportation, social isolation, and pathogens, and internal factors, such as so-called oxidative stress. It is necessary to evaluate the degree of stress responses due to its deleterious consequences on the production, reproduction, and welfare of animals [9]. Oxidative stress is considered to be an important factor leading to aging, inflammation, and even disease. Inflammation is a host’s defensive pathological response to various physical, chemical, biological, and other harmful stimuli. Acute inflammation is an immediate and adaptive response to infectious agents, and it induces the immediate recruitment and activation of natural killer cells, neutrophils, and eosinophils, while chronic inflammation gives rise to considerable damage to tissues, which may cause procarcinogenic conditions [10,11,12]). Moreover, as OS can gradually cause chronic inflammation by activating various transcription factors, such as peroxisome proliferator-activated receptor γ, nuclear factor kappa-B, and activator protein-1, it can be reciprocally connected to inflammation [12]. In mammals, it is well known that, as newborns begin breathing via the so-called induction of pulmonary respiration, they experience oxygen-rich air for the first time, which increases the production of ROS, particularly in lung cells [13,14]), and as the level of ROS goes beyond the antioxidant capacity in neonatal ruminants, OS can gradually develop [15]. In many countries, newborn dairy calves are susceptible to infectious diseases, so their morbidity and mortality rates are commonly higher during their first month of life [16,17]). The high disease susceptibility of neonatal calves is attributed to their low ability to establish an effective immune system against pathogens, due, in part, to the relatively low response of calf lymphocytes to stimuli [18,19]). For this reason, neonatal ruminants need to consume colostrum as a source of immunoglobulin G in order to acquire passive transfer immunity in the first few days after birth, as colostrum with a high total antioxidant content, including enzymatic components (such as superoxide dismutase, lactoperoxidase, glutathione peroxidase, and catalase) and non-enzymatic components (provitamin β-Carotene, provitamin α-Tocopherol, vitamin C, lactoferrin, selenium, etc.), can provide protection against OS-induced damage [14,20]. However, in the past, when young ruminants were deprived of colostrum and completely weaned, they were administered antibiotics or some antioxidants (vit-C, vit-E, and selenium) to counteract OS deriving from excessive free radicals.

The United States have only banned antibiotics that are important for human medicine, and several antibiotics are still used as feed additives for animal feeding in the US, as they are favorable for promoting animal growth [21]. As the problem of bacterial resistance to antibiotics became a burning issue throughout the world, China also stated that antibiotics would be banned in animal feed from 2020. Previously, the most commonly used approaches to alleviate stress included the prevention of the accumulation of peroxides, the elimination of free radicals in the body, the regulation of carbohydrate metabolism, the relief of stress, and the consumption of antioxidants (such as vitamins C and E and beta carotene) [22,23]). However, most approaches can only temporarily alleviate symptoms rather than fundamentally treat diseases. The use of steroidal and non-steroidal anti-inflammatory drugs has been reported to be a highly effective treatment approach against inflammatory diseases, but other studies have reported that the long-term consumption of these drugs may have serious adverse effects on some animal organs, such as the gastrointestinal tract, the cardiovascular system, and the liver [24]. In order to sustain productivity and animal health under the antibiotic-free scheme, researchers have been exploring sustainable alternatives that have antimicrobial, antiparasitic, and immunomodulation properties but do not create resistance, cause adverse side effects, or leave residuals in the final products [25,26]).

Not only do plants contain the principle nutrients (fats, proteins, and carbohydrates), but they also contain secondary metabolites; these secondary metabolites play an important role in signaling connections between plants and their environment, contribute to defense approaches, give plants their taste and color, etc. [27]. These compounds, namely, plant extracts, ensure the directional acquisition and enrichment of one or a group of bioactive compounds with plants as raw materials through appropriate separation and extraction processes [28], and their use is commonly reported to be associated with health benefits due to their anti-inflammatory and antioxidant activities [29].

## 3. Plant Extracts and Their Anti-Inflammatory and Antioxidant Effects

Plant extracts have been used as dietary additives with high efficiency and safety throughout history due to their medicinal properties in enhancing animal health and productivity; they can function as plant-derived antioxidants that are bioactive substances with special functional groups.

### 3.1. Benefits of Plant Extracts Used as Feed Additives

Due to the implementation of the antibiotic banning policy, the livestock industry has begun to actively explore alternatives to antibiotics in animal feed, among which plant extracts are one of the important choices. Although people commonly favor the use of plants, the legalization of plant medicine is relatively slow for various reasons. Since 2012, with the gradual introduction of the restriction policy of antibiotics, plants, as one of the alternatives to antibiotics, have received support from the Ministry of Agriculture and Rural Affairs of China for their inclusion in policies. At present, in China, there are 117 kinds of natural plants in the Catalogue of Feed Materials that do not endanger animal health or food safety [30]. In the past decade, the application of plant extracts in the prevention and recovery of animal health issues, the reproduction of livestock, and the reduction of methane in the rumen, plus their use as both growth promoters and immune boosters, have increased due to the development of green livestock production systems and the concern regarding toxic residues in food [31]. Moreover, plant extracts have been used due to the failure of modern drug therapies against chronic diseases, their side effects, and microbial resistance [32]. According to the effects of plant extracts in animal rearing, we can summarize the advantages of their application in the livestock industry as follows: (1) Improving feed safety: plant extracts have become the best choice to replace the partial use or complete use of antibiotics, without causing resistance or side effects. (2) Creating product differentiation: under the market background with serious homogenization, plant extracts can help feed companies to create high-quality and differentiated commercial feed by taking advantage of replacing antibiotics and repelling insects as well as improving immunity, reproductive performance, and meat quality. (3) Scientific and accurate efficacy: compared with crushed ingredients, plant extracts have clear components and mechanisms of action, allowing for more accuracy in feed production and animal rearing.

### 3.2. Modes of Action of Plant Extracts

Oxidative stress, even subsequent inflammation, is known to be an important factor in the development of chronic degenerative diseases, including atherosclerosis, coronary heart disease, gastrointestinal diseases, arthritis, neurodegenerative diseases, cancer, and diabetes [33,34]). Thus, it can be regarded as a concomitant reaction of most diseases. Correspondingly, controlling OS or inflammation is a critical strategy of maintaining well-being during breeding. At present, it is believed that plant extracts mainly control OS through four modes of action.

First, the antioxidant components of plant extracts have the ability to donate hydrogen atoms to metals, which restricts pro-oxidative activity [35]. Phenolics are known to be effective antioxidants, the efficacy of which has been considered to be more potent than that of vitamins E and C [36]. The inverse correlation between the intake of vegetables and fruit and the risks of cancer, diabetes, and cardiovascular and neurodegenerative diseases has partially been attributed to phenolics [37,38]). *Proanthocyanidins*, one of the polyphenol compounds that are abundant in bearberry and green tea, has been proven to donate hydrogen atoms/electrons and to function as an antioxidant [39,40]). Second, with more hydroxyl groups present in the skeletons of plant extracts such as flavonoids, the plant extracts can potentially possess a higher capacity of antioxidants because they can provide more electrons [41]. Many studies have reported that phenolic compounds with higher hydroxylation, particularly those located ortho to one another (catechol moiety, namely, vicinal OH groups), exert extremely high antioxidant activities [42,43]). Quercetin has been reported to be a flavonoid possessing a high antioxidant capacity because it has hydroxyl groups and a twisting angle of the B ring [44]. In the planar structure of quercetin, the methylation of hydroxyl groups in the B ring has been found to decrease the antioxidant ability the most [44]. Third, plant extracts can improve the antioxidant capacity in animal tissues by reducing oxygen concentrations and quenching oxygen, thus preventing peroxide production while activating antioxidant enzymes. Flavonoids can activate phase II detoxifying enzymes, such as glutathione S-transferase, NAD(P)H-quinone oxidoreductase, and UDP-glucuronosyl transferase, which are the main enzymes that defend against OS and electrophilic toxicants [45]. These defensive gene expressions can be regulated by an electrophile responsive element (EpRE), which is a regulatory sequence of genes responsible for encoding these phase II enzymes [46]. Lastly, other modes of action have been explored to address the antioxidant capacity of plant extracts, such as interactions with specific proteins central to intracellular signaling cascades [47], the modulation of the expression and the activity of key proteins [48], the effects on epigenetic mechanisms [49], and the modulation of the gut microbiota [50]. Fermented foods commonly attract consumers’ attention due to the fact that they have an improved shelf life and flavor as well as containing various health-enhancing compounds [51]. Tonolo et al. [52] found four peptides (i.e., N-15-M, E-11-F, Q-14-R, and A-17-E) in fermented milk produced using various *Lactobacillus*, and they identified that these bioactive peptides can act as antioxidants via the activation of the Keap 1/erythroid 2-related factor 2 (Nrf2) pathway. Cyanidin-3-glucoside is known to be metabolized in the gastrointestinal tract, where it produces various secondary phenolic compounds, such as phloroglucinaldehyde, protocatechuic acid, ferulic acid, and vanillic acid [53]. Tan et al. [54] reported that these metabolites can exert various effects by modulating the gut microbiota and by regulating Nrf2 antioxidant and inflammatory pathways.

## 4. Mechanisms of Representative Functional Plant Extracts as Antioxidants

Plant-derived antioxidants mainly come from herbs, spice plants, fruits, and vegetables, and according to their different chemical structures and biological properties, plant extracts are commonly categorized as polyphenols, polysaccharides, saponins, alkaloids, etc. [55,56,57,58].

### 4.1. Polysaccharides

**Figure 1:**
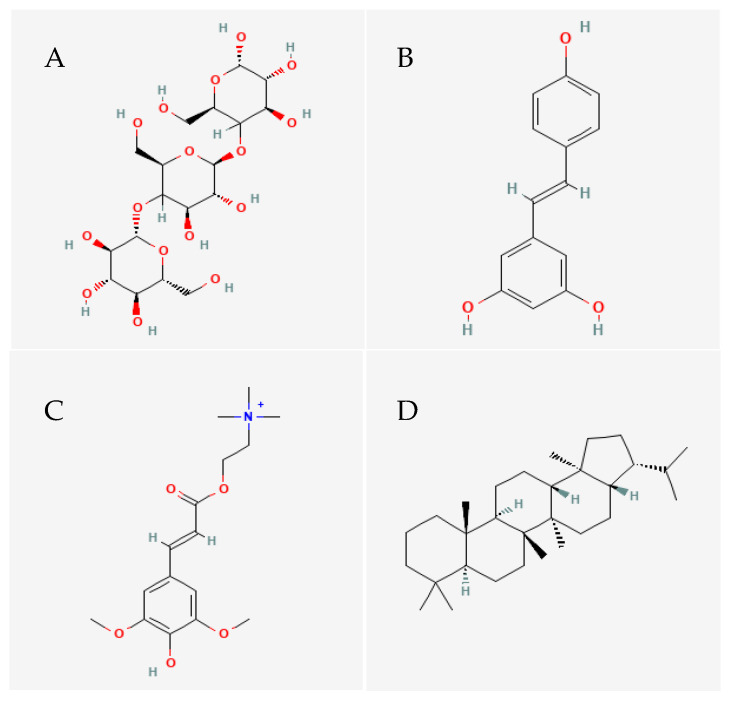
Chemical structure depiction of four representative functional plant extracts: (**A**) β-glucan; (**B**) resveratrol; (**C**) sinapine; (**D**) hopanes (reproduced with permission from [63]).

Plant polysaccharides are commonly known to be functional compounds, such as pectin and β-glucan (Figure 1A), the backbone of which is composed of α−1, 4, α−1, 6, β−1, 3, and β−1, 4 glycosidic linkages [59]. Many studies have identified their pharmacological and biological activities, such as their antioxidant, anti-inflammatory, immunomodulatory, and antitumor activities [60,61]. It has been reported that polysaccharides as antioxidants have various mechanisms of action, including the regulation of antioxidant enzymes via the Nrf2/ARE pathway, the removal of free radicals, and the antagonizing of nitric oxide (NO) [62] (Table 1).

Superoxide dismutase (SOD), catalase (CAT), and glutathione peroxidase (GSH-Px) constitute the enzyme antioxidant system, and the expression levels of these enzymes are mainly modulated by nuclear factor-E_2_-related factor 2 (Nrf2), which is an important factor of antioxidative stress [64]. Under normal conditions, Nrf2 can combine with Kelch-Like ECH Associated Protein 1 (Keap1) and degrade and return to a low level in the cytoplasm, while under OS conditions, Nrf2 can be released from Keap1 and move to the nucleus, where it combines with antioxidant response element (ARE) and then modulates the expressions of antioxidant proteins and phase II detoxification enzymes [65]. Polysaccharides are known to improve the expressions of antioxidant genes and proteins via the Nrf2-ARE pathway. Sun et al. [66] reported that polysaccharides from *Astragalus* could enhance myocardial antioxidant activity and mitigate OS in rats with arthritis through the regulation of the Keap1/Nrf2-ARE signaling pathway.

Free radicals are an active atomic group, and, therefore, they persistently catch other electrons to maintain stable conditions. Under a balanced state of the production and elimination of free radicals, free radicals generated via oxidation can modulate cell generation and the signal transmission of cells and inhibit the invasion of viruses and bacteria into human and animal bodies. However, due to the fact that the body is affected by internal activities and subjected to stress from external factors and emotional fluctuations, it is difficult to maintain a balance between the generation and clearance of free radicals, leading to OS. Polysaccharides have been reported to modulate and improve the capacity of antioxidant enzymes and to eliminate free radicals [67]. Zhu et al. [68] reported that extracellular polysaccharides from *Cordyceps militaris* could effectively eliminate hydroxyl free radicals, superoxide anion free radicals (O_2_^•−^), and 1,1-diphenyl-2-trinitrophenylhydrazine (DPPH) free radicals, and that antioxidant activity could, to some extent, be enhanced with an increase in the dose of polysaccharides.

Nitric oxide is also an active and unstable molecule, the generation of which depends on the existence of a family of three nitric oxide synthetase (NOS) enzymes: neural NOS (nNOS), endothelial NOS (eNOS), and inducible NOS (iNOS) [69]. Under normal conditions, NO has neuroprotection and neurotoxic functions, and when overproduced due to the overexpression of iNOS, NO can cause the generation of abundant free radicals, including NO_2_ free radicals and hydroxyl free radicals, leading to the damage of cell membranes, mitochondria, proteins, and nucleic acid, and, furthermore, accelerating cell apoptosis [70]. For these reasons, it is necessary to appropriately inhibit the overproduction of iNOS and subsequent NO to mitigate OS. Han et al. [71] reported that polysaccharides from *Acanthopanax senticosus* could modulate the expressions of iNOS proteins to limit the overproduction of NO, exerting a protective influence on the oxidative injury of hippocampal neurons.

### 4.2. Polyphenols

In plants, most polyphenol compounds, such as resveratrol (Figure 1B) and proanthocyanidins, have antioxidant effects. By scavenging free radicals in the body, they can improve the immune function of the body and mitigate oxidative damage in animals. It has been reported that polyphenols as antioxidants have various mechanisms of action, which can mostly be divided into specific and non-specific actions [72].

The non-specific actions are achieved by scavenging free radicals and chelating metal due to the existence of two or three double bonds and the 4-keto group in ring C and the existence of phenolic groups [72]. Kruk et al. [73] summarized the non-specific protective mechanisms of polyphenol compounds: they can interact with membranes via both hydrophilic and hydrophobic interactions, which allows them to be positioned at different membrane levels; one such position is on the polar head groups of phospholipids in membranes, generating hydrogen bonds, leading to insertion into lipid bilayers and then interactions with the hydrophobic part of the lipid chain. Using this mechanism, polyphenols can protect membranes and their components from oxidative injury.

The specific actions are connected to the conformational and structural characteristics of polyphenols and their interactions with membrane components, including lipids and proteins [72]. Briefly, the specific mechanisms are involved in interactions with specific proteins, and the effects depend on the functions of these proteins and the metabolites of their biotransformation [73]. This kind of mechanism incorporates interactions with enzymes related to inflammation, including 5-lipoxygenase (LOX), cyclo-oxygenase enzymes 1 and 2 (COX 1/2), and phospholipase A2 (PLA_2_), and it includes reductions in O_2_^•−^ levels and NO• availability and the modulation of blood pressure. Another specific mechanism incorporates the regulation of redox-sensitive transcription factors, such as transcription factor activator protein (AP-1) and nuclear factor kappa B (NF-κB) (related to intracellular signaling cascades), or interactions with estrogen receptors, functioning as estrogen antagonists or agonists, owing to the similar structures of isoflavones and estrogens [74]. Yahfoufi et al. [75] reported that the application of polyphenols could inhibit markers of OS, namely, secondary products of the inflammation response to other stressors.

### 4.3. Alkaloids

Alkaloids, such as sinapine (Figure 1C) and morphine, exist in about 20% of plant species. Alkaloids are complex organic compounds with nitrogen ring structures, having both antioxidant and pro-oxidant effects depending on the conditions [76]. Due to the very diverse structures of alkaloids, their number is very extensive, and there are up to 16,000 structures [77]. As the ROS/antioxidant system is complex, the mechanisms of action that alkaloids exert against OS remain unclear, and the possible pathways determined to mitigate OS to date can be summarized as follows: indicaxanthin and aloperine can inhibit the enzymatic subunit synthesis of the NADPH-oxidase complex, which is an important enzyme for reactive oxygen species generation; some compounds, such as cepharanthine, capsaicin, and evodiamine, can inhibit the phosphorylation of NADPH-oxidase complex subunits; morphine, boldine, and sinomenine can inhibit the production of the active NADPH-oxidase complex; some compounds, such as betanin, coptisine, and berberine, can activate Nrf2; other compounds, including Dendrobium alkaloids, dehydropipernotaline, and harmine, can activate some nuclear factors exerting antioxidant effects (e.g., PPAR and FOXO); eurochevalierine, berberine, mahanine, etc., can be involved in epigenetic effects, including DNA methylation, histone methylation or acetylation, and the expression of miRNA; and harmaline can directly inhibit myeloperoxidase [76].

### 4.4. Saponins

Saponins, such as hopanes (Figure 1D) and cycloartanes, are compounds with complex structures, and they are composed of sapogenins, sugar chains, and organic acids. The type of saponin depends on the position, number, and mode of the sugar chains, including a large family of amphiphilic glycosides of steroids and triterpenes. According to the various saponin structures, saponins express a wide range of pharmacological and biological characteristics and function as important active components in folk medicines [78]. As for the anticancer, anti-inflammation, and antioxidation effects of saponins, the findings of some researchers can be summarized as follows. Some compounds, such as saponins from plant extracts, exert cytotoxicity effects against cancer cells, such as HTC-8 (human colon carcinoma), BEL-7402 (human liver carcinoma), and A549 (human lung carcinoma) [79], which may be attributed to the effects of proapoptosis in the cancer cell lines [80]. Moreover, saponins have been found to inhibit the expressions of the VE-cadherin and VEGFR-2 complexes, which are key growth factors for vascular remodeling and proliferation via NF-κB downregulation [80]. Regarding antioxidation effects, saponins exert radical scavenging effects, in turn inhibiting lipid oxidation by breaking chains; therefore, they can function as nutraceuticals and pharmaceuticals [80]. Moreover, saponins can influence hydrogen peroxide-induced ROS production in the liver due to the fact that saponins can activate the expressions of the transcription factors of antioxidant enzymes, such as glutamate cysteine ligase (GCL) and NRF2 [81], thereby decreasing oxidation stress. Regarding anti-inflammation effects, saponins can mitigate inflammation by blocking the NF-κB signaling pathway. Fan et al. [82] reported that saponins, such as melittin, alleviated liver failure in D-galactosamine/LPS-induced mice by inhibiting the apoptotic pathway and the NF-κB signaling pathway.

## 5. Application of Plant Extracts in Ruminants

Compared to traditional chemical drugs, plant extracts are unique in controlling inflammation and OS. Furthermore, they comprehensively improve the health conditions of animals by improving their own immunity, achieving healthy breeding standards that prevent animal diseases and improving both animal performance and animal product quality [83].

### 5.1. Cattle

A large number of studies have been conducted to evaluate the effects of plant extracts on the growth performance, feed efficiency, and antioxidant capabilities of cattle (Table 2). Dietary supplementation with a composite feed additive consisting of a blend (1:1) of eucalyptus (*Eucalyptus citriodora*) and poplar (*Populus deltoids*) leaves containing total phenolics, tannin phenolics, and condensed tannins at two different dose levels (50 g and 150 g/h/d) did not affect growth performance or nutrient digestibility, but it did improve antioxidant status and immunity, and it reduced methane production in buffalo calves compared to a control group [84]. Regarding the improved immunity of the animals, the authors explained that the probiotic effects of the combined supplementation of both extracts containing essential oils and tannins could indirectly enhance the immune system by inhibiting hyper-ammonia-producing and proteolytic bacteria with the stimulation of *Bifidobacterium* and *Lactobacillus*. The effect of the combination of the extracts, that is, the increased antioxidant status, could be attributed to the existence of various bioactive compounds that can enhance the activity of antioxidant enzymes. Moreover, the mitigation of methane was connected to the regulation of the rumen microbiome via the regulation of either the inhabitation of methanogens or methanogenic archaeal diversity. Xu et al. [85] evaluated the effect of gallic acid on calves, reporting that gallic acid supplementation in starter feed improved growth performance parameters (such as average daily gain), rumen fermentation parameters (such as total volatile fatty acids, propionate, and butyrate), and antioxidant levels (such as catalase and T-AOC levels), but it decreased malondialdehyde and tumor necrosis factor-α levels in preweaning dairy calves. In addition, gallic acid supplementation increased the ruminal abundances of *Prevotellaceae_UCG-001*, *Saccharofermentans*, and *Prevotella_1*, but it reduced the abundance of *Prevotella_7*. Gallic acid, existing in plant extracts and belonging to phenolic compounds, has been reported to possess high antioxidant capabilities, as it has a scavenging effect on hydroxyl radicals and a strong reducing power [86]. Engler et al. [87] reported that feeding rumen-protected grape extract to vaccinated Prim’Holstein heifers could improve their overall antibody response to bovine respiratory syncytial virus and para influenza 3 virus as well as their total antioxidant capacity, which is attributed to the inclusion of grape polyphenols that can modulate superoxide dismutase expression and increase the total antioxidant status [88]. Silva et al. [89] compared the effects of feed additives on the nutrient intake, rumen fermentation, and milk composition of mid- to late-lactating Jersey cows—among saponins alone; saponins combined with essential oils containing carvacrol, cinnamaldehyde, and limonene; and monensin alone—and they found that the cows fed monensin supplements showed a lower dry matter, protein, and ether extract intake, whereas the cows fed saponins combined with a blend of natural essential oils showed higher milk fat and protein levels and ruminal butyrate concentrations compared to the cows fed monensin. The author explained that the increased fat content in the milk of the cows fed the saponins combined with the essential oils may be attributed to the increased ruminal butyrate production. A previous study also reported that butyrate metabolism in the ruminal epithelium is connected to beta-hydroxybutyrate production, which is associated with the synthesis of fatty acids in mammary glands and adipose tissue [90]. A previous study that evaluated the effects of grape seed extract supplementation on antioxidant activity and the inflammatory response in heat-stressed calves reported that diets with a supplementation of 50 and 100 mg/kg of BW/day improved the plasma levels of TAC and SOD, the hematocrit percentage, red blood cells, and hemoglobin values while decreasing the plasma levels of MDA and TNF-α and the neutrophil count in the heat-stressed calves compared to a control diet, indicating that grape seed extract can enhance the anti-inflammatory and antioxidant capacities of calves against oxidative and heat stress [91]. Grape seed extract is known to contain abundant phenolic compounds, such as gallic acid, epicatechin, catechin, and procyanidins, which can scavenge some free radicals, including superoxide, hydroxyl radical, hydrogen peroxide, and chelating iron, to decrease oxidant production and, in turn, exert antioxidant effects [92]. Moreover, grape seed procyanidins have been reported to decrease the concentrations of proinflammatory cytokines, such as TNF-α, IL6, and IFN-γ, by inhibiting NF-κB activity in the liver, which indicates that grape seed extract can also exert anti-inflammation effects [93].

In addition, Aguilar-Hernández et al. [94] reported that the use of alkaloids (isoquinoline alkaloids, such as sanguinarine and quelertrine) at a daily dose of 18 mg in steers fed high-grain diets improved protein utilization by reducing protein degradation in the rumen and by increasing microbial protein synthesis. Other effects were shown, such as an increase in ruminal molar proportion of acetate and a decrease in ruminal protozoa count [94,95]). It is well known that a high ambient heat load (HAHL) can cause oxidative stress, compromising animal productivity. Mendoza-Cortéz et al. [96] tested the effects of a supplemental blend of essential oils (thymol, eugenol, vanillin, guaiac, and limonene plus 25-hydroxy-Vit D3 dosed at 119 mg/kg DM) on the performance of cattle under HAHL (THI > 82) compared to the effects of sodium monensin (dosed at 24 mg/kg DM), and the results showed that supplementation with the blend of EO plus Vit D3 reduced the deleterious effects of HAHL, increasing daily weight gain by 8.3%, which was attributed to an increase in dietary energy utilization by 3% when compared to the steers supplemented with sodium monensin. de Zawadzki et al. [97] evaluated the effect of mate (*Ilex paraguariensis* A.St.-Hil.) extract on beef quality, and the results showed that the supplementation of mate extract at levels of 0.5, 1, and 1.5% w/w to the basal diet increased the contents of inosine monophosphate, creatine, and carnosine in the fresh meat. The total conjugated linoleic acid content in the meat increased, but the radical formation in the meat tended to decrease with the increase in the mate extract content in the feed. Gobert et al. [98] reported that the supplementation of Vit E and plant extracts rich in polyphenols added to PUFA-rich cow diets improved lipid stability in steaks, even in the most deleterious packaging systems, and the plant extracts preserved Vit E from depletion. Pena-Bermudez et al. [99] found that the inclusion of yerba mate (Ilex paraguariensis ST. Hilaire) at levels of 0.5, 1, and 2% of DM to the basal diet of Nellore steers did not affect the total apparent digestibility, rumen fermentation parameters, or ruminal CH4 emissions; however, ruminal NH3-N content was decreased as the extract level was increased in the diet. Prommachart et al. [100] reported that the inclusion of black-rice- and purple-corn-extracted residue (anthocyanin and phenolic acids) at 2, 4, and 6% of DM to the TMR of dairy cattle did not influence the intake and digestibility of most nutrients or the levels of most plasma parameters, but it did increase ether extract intake and decrease plasma MDA levels. Li et al. [101] found that the supplementation of mulberry leaf flavonoids at doses of 15, 30, and 45 g/d to the TMR of Murrah buffaloes decreased serum MDA content while increasing serum heat shock proteins and GSH-Px content. Moreover, 45 g/d of mulberry leaf flavonoids increased milk yield and the contents of fat-corrected milk and milk proteins, indicating that inclusion at 45 g/d could enhance milk performance and mitigate heat stress in buffaloes. Vizzotto et al. [102] reported that the supplementation of oregano (containing the essential oil of oregano plants) at 10 g/d and green tea extracts (containing polyphenols) at 5 g/d to the basal diet of Jersey cows increased the total digestive nutrients and ME intake, but it reduced the level of reactive species in erythrocytes. Moreover, they further increased the level of reduced glutathione in the plasma, overall indicating that the extracts reduce some oxidative stress biomarkers in plasma. Some studies have also evaluated the antioxidant effects of plant extracts in animals in vitro models of oxidative stress. Ciampi et al. [103] observed the antioxidant effects of pomegranate (*Punica granatum*), tara (*Caesalpinia spinose*), chestnut (*Castanea sativa*), and gambier (*Uncaria gambir*) natural extracts using an in vitro bovine aortic endothelial cell model of oxidative stress, and they found that the addition of chestnut and tara extracts at 80 μg/mL decreased endothelial cell viability and that pomegranate decreased the production of isoprostane, which is a product of lipid peroxidation following the induction of oxidative stress. Moreover, intracellular ROS production was highly decreased in the cells treated with natural extracts compared to the cells treated with lipopolysaccharides as a control, indicating the strong antioxidant activity of natural extracts. Sun et al. [104] reported that the supplementation of dandelion aqueous extract at 10 and 50 μg/mL to the bovine mammary epithelial cell line MAC-T suppressed the adverse effects of LPS-induced oxidative stress via the scavenging of cellular ROS and improvements in antioxidant enzyme activity. It also improved the upregulation of antioxidant gene expressions by activating the Nrf2 signaling pathway.

### 5.2. Sheep and Goats

The application of plants or their extracts as feed components or feed additives has also been evaluated in sheep and goats (Table 3). In a previous study, a diet with mulberry leaf silage as a new feed resource, replacing alfalfa silage, improved the antioxidant capability and immunity of lambs and correspondingly enhanced their health by enhancing the abundance of *Bifidobacterium* and *Lactobacillus* in the rumen while inhibiting *Ruminococcaceae UCG-010* and *Lachnospiraceae_XPB1014 group* [105]. These beneficial effects were attributed to the mulberry leaf’s content of polyphenols and polysaccharides, as these bioactive compounds can mitigate inflammation processes by enhancing serum IFN-γ levels and influencing the microbiota [106]. In a previous study, four plant extracts (rosemary, grape, citrus, and marigold) rich in polyphenols were evaluated for their bioavailabilities and antioxidant capacities in sheep, and the results showed that the plant extracts, especially grape, could effectively improve the plasma total antioxidant status and that marigold decreased plasma susceptibility to lipoperoxidation, confirming that polyphenols were attached at the aqueous–lipid interface or to the lipid structure, as they could not quench lipoperoxides by linking with proteins [107]. Mu et al. [108] tested the effects of grape seed procyanidins as antioxidants in ruminants, and they found that the grape seed procyanidins decreased the levels of proinflammatory cytokines, such as IL-1β and TNF-α, in the serum and colons of lambs, while they increased the activities of GSH-Px and SOD in the serum and the levels of GSH-Px and T-AOC in the colonic mucosa tissue. This resulted in the alleviation of the inflammatory response in the colon epithelium induced by the highly concentrated diet, as antioxidant metabolism and energy and amino acid metabolism were improved, and as arachidonic acid metabolism was mitigated. As for the biological function of procyanidins, many studies have been conducted, and they reported that procyanidins could induce Nrf2 nuclear translocation to improve the Nrf2 signaling pathway and, in turn, increase the expressions of downstream target genes, such as GSH-Px and SOD, leading to the mitigation of OS in animals [109,110]. In addition, procyanidins are known to mitigate inflammation by directly regulating cellular signaling pathways, thereby exerting strong anti-inflammatory activities [111]. Zhang et al. [112] examined the antioxidant effects of polysaccharide-rich noni (*Morinda citrifolia* L.) fruit extract in goats, and they found that dietary supplementation with 0.4% of noni fruit extract improved the concentrations of TNF-α and IL-6 and the activities of T-SOD, TrxR, GPx, and catalase in the serum, while it reduced the levels of ROS and MDA in the serum of cashmere goats. GPx1, a GPx family member, is known to reduce hydroperoxides to relevant alcohols using glutathione as a cofactor, and GPx4 can act as an antioxidant enzyme to reduce the hydroperoxides of phospholipids in lipoproteins and membranes [113]. Moreover, the antioxidant capacity of noni fruit extract may be attributed to the number of hydroxyl chelated with metal ions in polysaccharides, as chelates play important roles in reducing lipid peroxides and scavenging free radicals [114]. Studies have also evaluated the antioxidant effects of plant extracts on animal products. Luo et al. [115] investigated the antioxidant effects of *Astragalus membranaceus* supplementation on goat muscles. The results showed that *Astragalus membranaceus* supplementation increased myoglobin content and the activities of some antioxidant enzymes (SOD and catalase), and it improved the values of free radical scavenging ability and cupric reducing antioxidant capacity in goat muscle, but it decreased the contents of metmyoglobin and MDA, indicating an enhanced antioxidant capacity in the goats. The authors elucidated that, as a high myoglobin content is due to a high ratio of type I fiber, the high myoglobin content reflected a higher CIE a* (redness degree) value of the muscle [116], indicating less lipid oxidation in the muscles of the goats fed *Astragalus membranaceus*. The radical scavenging ability indicated the free-radical-scavenging ability of all the antioxidant components in the muscle tissue, which was attributed to the abundant antioxidant-containing components (polyphenol, flavonoid, and polysaccharide compounds) in *Astragalus membranaceus* [117].

In addition, Estrada-Angulo et al. [118] reported that the supplementation of a mixture of quaternary benzophenanthridine alkaloids and protopine alkaloids to ewes under HAHL improved the utilization efficiency of dietary energy by 5.2%, and it reduced the scores for cellular dropsical degeneration, neutrophilic infiltration, and parakeratosis in ruminal epithelia cells. Wang et al. [119] found that the supplementation of wheat bran feruloyl oligosaccharides at 100 and 200 mg/kg of feed in the diet of lambs improved ADG, the feed conversion rate, serum T-AOC levels, antioxidant enzyme activity, and glutathione content, while it decreased ruminal NH3-N content. Damiano et al. [120] reported that supplementing 90 mg/kg of red orange and lemon extract to the diet regimen of lambs improved the activities of SOD, CAT, and GPx in the plasma, while it decreased the levels of MDA, 8-hydroxy-2′-deoxyguanosine, IL-1β, and IL-6 in the plasma. Beck et al. [121] found that the addition of *Lactobacillus* fermented plant extracts with terrestrial plant extracts (chicory, plantain, lucerne, and leaf dock) at 10 mL/d to the diet of ewes did not affect the plasma levels of the total antioxidant status or the GPx of the ewes, but it did improve the total antioxidant status level and decrease GPx activity in the plasma of their lambs, indicating that the plant extracts increased the maternal transmission of antioxidants from the ewes to their offspring. Wang et al. [122] reported that adding 0.3% of fermented wheat bran polysaccharides to the diet of lambs increased ADG and the apparent digestibility of DM, OM, and CP. Moreover, it improved the plasma levels of IgG, IgM, and IL-10 and CAT activity. Li et al. [123] reported that the supplementation of a combination of nano-ammonium octamolybdate (nano-mo; 10 mg/kg of DM) and *Macleaya cordata* extracts (MCE; 3000 mg/kg of DM) to the diet of goats decreased Cu content, but it increased Fe and Mo contents in the serum and the liver of grazing goats exposed to grasslands contaminated with heavy metal. Moreover, this combination increased hemoglobin; erythrocyte count; packed cell volume levels; and the activities of SOD, GSH-Px, CAT, plus ceruloplasmin in the serum. However, it decreased blood white blood cell content and serum MDA content. In the studies evaluating the antioxidant effects of plant extracts on animal in vitro models of oxidative stress, Reyes-Becerril et al. [124] found that supplementing 150 μg/mL of oregano (*Lippia palmeri* Watts) extracts to goat peripheral blood leucocytes increased peripheral blood leukocyte content and improved nitric oxide production, phagocytosis, and SOD activities, whereas it downregulated proinflammatory (TNF-α and IL-1β) transcription and antioxidant (CAT and GPX-4) genes. In an in vivo test, supplementing 2.6% of oregano extract to the diet of goats increased caproic acid production and compounds related to the improvement of intestinal health, indicating the strong antioxidant capacity of oregano.

In this review, the varying degrees of the positive effects of plant extracts on animal performance, especially on antioxidant capacity, were identified not only in cattle but also in sheep and goats. Most of the mechanisms of action highlighted exerted antioxidant effects via direct actions, including the scavenging of free radicals, decreasing tissue susceptibility to lipoperoxidation, and decreasing hydroperoxides, or else via indirect actions, such as increasing the activities of antioxidant enzymes by modulating antioxidant-enzyme-related pathways, i.e., the Nrf2-ARE signaling pathway. In this review, we mainly summarized the comprehensive mechanisms of polysaccharides, polyphenols, alkaloids, and saponins published to date. There is still a need to explore their more precise mechanisms of action and novel bioactive compounds and to further verify their application effects on livestock.

## 6. Conclusions

Plant extracts have been widely recognized as potential functional alternatives to antibiotics due to their green, safe, and efficient properties. Compared with other alternatives (such as some vitamins alone) to antibiotics, plant extracts have the unique advantages of being natural and extensive sources, alleviating OS, and improving the immunity of animals and exerting positive effects on their meat products. However, there are still some shortcomings in the application of plant extracts in ruminants, including the limited production of extracts; undeveloped technology of extraction and processing; unclear appropriate dose levels depending on the species, age, and body weight of the animal; unstable effects; and high costs. In addition, the existence and mechanisms of action of many other plant extracts have not been explored, and corresponding animal feeding trials have not yet been carried out. Therefore, there is still much room and many opportunities for the development of plant extracts in the future.

## Figures and Tables

**Table 1:** Four representative plant extracts and their potential action mechanisms on both oxidative stress and inflammation related pathways.

Type	No.	Pathways	Mechanism
Polysaccharides	1	Regulation of antioxidant enzyme via Nrf2/ARE pathway	Under oxidative stress state, Nrf2 can be released from Keap1, move to nucleus, where it combines with ARE, and then modulates the expression of antioxidant protein and phase II detoxification enzyme. Polysaccharide can improve the expression of antioxidant gene and protein via Nrf2-ARE pathway [62].
	2	Removal of free radical	When animals are exposed to stress, it is difficult to hold the balance of generation and clearance of free radicals by themselves. Polysaccharide can modulate and improve the capacity of antioxidant enzymes and eliminate the free radicals [62].
	3	Antagonizing nitric oxide	Under the state of over-production of NO due to over-expression of iNOS, NO can cause the generation of abundant free radicals, leading to the damage of cell components. Polysaccharide can modulate the expression of iNOS protein to limit the over-production of NO [62].
Polyphenols	1	Specific interaction	The specific mechanisms are involved in interactions with specific proteins, and the effect depends on the functions of these proteins and metabolites of their biotransformation. This kind of mechanism incorporates interactions with enzymes related to inflammation, including LOX, COX 1/2, and PLA2, and includes a reduction of O_2_^•−^ level and NO• availability, and modulation of blood pressure [73].
	2	Specific interaction	Another specific mechanism incorporates regulation of redox-sensitive transcription factors, such as AP-1 and NF-κB, or interaction with estrogen receptors, functioning as estrogen antagonists or agonists, owing to similar structure between isoflavones and estrogens [73].
	3	Non-specific interaction	Polyphenol compounds can interact with membranes owing to both their hydrophilic and hydrophobic interactions, which allows the polyphenol compounds to be positioned at different membrane levels, one of which is that polyphenol is placed on the polar head groups of phospholipids in membrane, generating the hydrogen bonds, leading to insertion into lipid bilayers, and then interacting with the hydrophobic part of the lipid chain. Through this mechanism, polyphenol can defend membranes against oxidative injury [73].
Alkaloids	1	Inhibition of NADPH-oxidase complex	Indicaxanthin and aloperine can inhibit the enzymatic subunit synthesis of NADPH-oxidase complex that is an important enzyme for reactive oxygen species generation [76].
	2	Inhibition of production of active NADPH-oxidase complex	Morphine, boldine, and sinomenine can inhibit the production of active NADPH-oxidase complex [76].
	3	Inhibition of the phosphorylation of NADPH-oxidase complex	Some compounds such as cepharanthine, capsaicin, evodiamine, etc. can inhibit the phosphorylation of NADPH-oxidase complex subunits [76].
	4	Activation of Nrf2 enzyme	Some compounds, such as betanin, coptisine, berberine, etc., can activate Nrf2 [76].
	5	Activation of antioxidant factors	Other compounds, including Dendrobium alkaloids, dehydropipernotaline, harmine, etc., can activate some nuclear factors exerting antioxidant effects (e.g., PPAR, FOXO) [76].
	6	Epigenetic effects	Eurochevalierine, berberine, mahanine, etc. can be involved in epigenetic effects, including DNA methylation, histone methylation or acetylation, and expression of miRNA [76].
	7	Inhibition of myeloperoxidase	Harmaline can directly inhibit myeloperoxidase [76].
Saponins	1	Radical scavenging effect	Saponins exert radical scavenging effect, in turn inhibiting lipid oxidation through breaking the chain, and therefore, can function as nutraceuticals and pharmaceuticals [80].
	2	Inhibition of the hydrogen peroxide-induced ROS production	Saponins can influence the hydrogen peroxide-induced ROS production in the liver due to the fact that saponins can activate the expression of the transcription factor of antioxidant enzymes, such as GCL and NRF2, thereby decreasing the oxidation stress [80].
	3	Inhibition of NF-κB signaling pathway	For anti-inflammation effects, saponins can mitigate inflammation through blocking the NF-κB signaling pathway [80].

Abbreviations: Nrf2, nuclear factor-E2-related factor 2; Keap1, Kelch Like ECH Associated Protein 1; ARE, antioxidant response element; iNOS, inducible nitric oxide synthetase; LOX, 5-lipoxygenase; COX 1/2, cyclo-oxygenase enzymes 1 and 2; PLA2, phospholipase A2; AP-1, activator protein 1; NF-κB, nuclear factor kappa B; NADPH-oxidase, nicotinamide adenine dinucleotide phosphate-oxidase; PPAR, peroxisome proliferators-activated receptors; FOXO, forkhead box transcription factors; GCL, glutamate cysteine ligase.

**Table 2:** Effects of plant extracts on growth performance, feed utilization, antioxidants capability, and animal products in cattle.

Plants or Plant Extracts	Dose Levels	Supplement Method	Animal Species	Gender	No. of Animal	Age	Body Weight	Findings
Blend of eucalyptus and poplar leaves	50 g, 150 g/h/d	Mixing with concentrate mixture	Murrah buffalo calves	Female	18	10–14 months old	131.68 ± 7.5 kg	Antioxidant status and immunity (↑); methane production (↓); growth performance and nutrient digestibility (ND) [84].
Gallic acid	0.5 g, 1 g/kg in starter diet	Adding to starter feed	Holstein calves	Female	36	3.1 ± 1.39 days old	40.8 ± 2.87 kg	Growth performance, plasma total protein and β-hydroxybutyrate levels, ruminal total VFA, propionate, and butyrate concentrations, and total antioxidant capacity (↑); malondialdehyde and tumor necrosis factor-α concentrations (↓); the ruminal abundances of *Prevotellaceae_UCG-001, Saccharofermentans*, and *Prevotella_1* (↑), the abundance of *Prevotella_7* (↓) [85].
Rumen-protected grape extract	670 mg/animal/d	Diluting to a mixture of calcium carbonate and crushed wheat, and top-dressed	Prim’ Holstein heifers	Female	22	6–8 months old	161 ± 17 kg	Overall antibody response to bovine respiratory syncytial virus and para influenza 3 virus, and animals’ total antioxidant capacity (↑) [87].
Saponin combined with essential oils	16 g/cow/day	Mixing with basal diet	Lactating Jersey cows	Female	8	NS	NS	Fat and protein levels in milk, and ruminal butyrate concentration (↑), as compared to cows fed monensin supplement [89].
Grape seed extract	50 and 100 mg/kg of BW/day	Adding into milk	Newborn Holstein calves	Female	60	3 days old	40.6 ± 2.17 kg	Plasma levels of TAC and SOD, and hematocrit percentage, red blood cells, and hemoglobin value (↑); plasma levels of MDA, TNF-α, and neutrophil count (↓) [91].
Quaternary benzophenanthridine alkaloids and protopine alkaloids	6, 12, 18 mg/steer/day	Adding into basal diet	Holstein steers	Male	4	NS	253 ± 9 kg	Protein utilization and microbial protein synthesis, and ruminal molar proportion of acetate (↑); ruminal NH_3_-N content (↓) [94].
Essential oils (EO: thymol, eugenol, vanillin, guaiac and limonene) plus Vit D3	119 mg of EO plus 0.12 mg of Vit D3	Adding into basal diet	Young bulls	Male	90	10 months old	228.0 ± 7.1 kg	Daily weight gain and gain/feed ratio (↑) [96].
Mate (*Ilex paraguariensis* A.St.-Hil.) extract (alkaloids, saponins, phenolic acids)	0.5, 1, 1.5% of DM	Mixing with basal diet	Nellore steers	Male	48	21 Months old	419 kg	Animal performance and carcass characteristics (ND); the levels of inosine monophosphate, creatine, carnosine, and CLA content, and tenderness and acceptibility of beef (↑); radical formation in meat (↓) [97].
Vit E plus plant extracts rich in polyphenols	7 g/kg of DM	Mixing with basal diet	Normand cull cows	Female	15	48~60 months old	649 ± 41 kg	Lipid stability in steaks (↑), even in the most deleterious packaging systems; plant extracts preserved Vit-E from depletion [98].
Yerba mate extract (Ilex paraguariensis ST. Hilaire)	0.5, 1, 2% of DM	Mixing with basal diet	Nellore steers	Male	8	NS	401.6 ± 32.3 kg	Total apparent digestibility, rumen fermentation parameters, and ruminal CH_4_ emissions (ND), except NH_3_-N [99].
Black rice and purple corn extracted residue	2, 4, 6% of DM	Mixing into TMR	Dairy cattle	Male	16	7~8 months old	160 ± 10.6 kg	Most of nutrients intake and digestibility, and most of plasma parameters levels (ND); ether extract intake (↑); plasma MDA level (↓) [100].
Mulberry leaf flavonoids	15, 30, 45 g/d	Mixing into TMR	Murrah buffaloes	Female	40	NS	600 ± 50 kg	Serum MDA level, T-AOC and CAT contents (↓); serum HSP and GSH-Px contents, GH and PRL hormones and E2 level (↑); milk yield, and serum T3 and T4 contents, and fat corrected milk and milk protein (↑), indicating treatments enhanced milk performance and mitigated heat stress [101].
Oregano and green tea extracts	10 g/d of oregano, 5 g/d of green tea extract	Mixing with basal diet	Jersey cows	Female	24	NS	441 ± 27 kg	Oregano: total digestive nutrients and ME intake, and carbonylated protein content (↑); the level of reactive species in the erythrocytes, and milk pH and somatic cell count (↓); Green tea: plasma eosinophils content (↑); the level of reactive species in the erythrocytes (↓); Both extracts: the level of reduced glutathione, overall BW (↑), indicating extracts reduced some oxidative stress biomarkers in plasma [102].
Pomegranate, tara, chestnut, gambier natural extract	80 μg/mL	NS	Bovine aortic endothelial cells (BAEC)	NS	NS	NS	NS	Chestnut and tara extracts: BAEC viability (↓); Natural extracts: intracellular reactive oxygen species production (↓), strongly showing antioxidant activity; Pomegranate: isoprostanes production (↓) that is the products of lipid peroxidation after induction of oxidative stress [103].
Dandelion aqueous extract	10, 50, 100, 200 μg/mL	NS	Bovine mammary epithelial cell line MAC-T cells	NS	NS	NS	NS	The adverse effect of LPS-induced oxidative stress (↓) via scavenging cellular ROS and improving antioxidant enzyme activity; the upregulation of antioxidative gene expression (↑) by activating Nrf2 signaling pathway [104].

NS: not shown; (↑): increase; (↓): decrease; ND: no difference. Abbreviations: BW, body weight; VFA, volatile fatty acid; TAC, total antioxidant capacity; SOD, superoxide dismutase; MDA, malondialdehyde; TNF-α, tumor necrosis factor-alpha; CLA, conjugated linoleic acid; HSP, heat shock proteins; GSH-Px, glutathione peroxidase; GH, growth hormone; PRL, prolactin; E2, estradiol; T3, tri-iodothyronine; T4, thyroxine; BAEC, bovine aortic endothelial cells; Nrf2, nuclear factor erythroid 2-related factor 2.

**Table 3:** Effects of plant extracts on growth performance, feed utilization, and antioxidant capability in sheep and goats.

Plants or Plant Extracts	Dose Levels	Supplement Method	Animal Species	Gender	No. of Animal	Age	Body Weight	Findings
Sheep								
Mulberry leaf silage	20% of DM	Mixing into TMR	Tan lambs (Ovis aries)	NS	40	75 ± 3 days old	15.3 ± 1.92 kg	Antioxidant capacity and immune function (↑); the abundance of *Bifidobacterium, Lactobacillus* and *Schwartzia* (↑); *Ruminococcaceae UCG-010* and *Lachnospiraceae_XPB1014_group* (↓) [105].
Rosemary, grape, citrus, marigold	10% of DM	Administration via rumen cannula	Castrated Texel sheep	NS	5	18 months old	50 ± 4 kg	Grape: plasma total antioxidant status (↑); Marigold: plasma susceptibility to lipoperoxidation (↓) [107].
Grape seed procyanidins	10, 20, 40 mg/kg of BW/day	Administration by manually feeding	Dorper × small thin-tailed crossed lambs	Male	48	145 ± 9 days old	28.9 ± 1.67 kg	Proinflammatory cytokine levels in the serum and colon of lambs (↓), such as IL-1β and TNF-α; activities of GSH-Px and SOD in the serum, levels of GSH-Px and T-AOC in the colonic mucosa tissue (↑) [108].
Quaternary benzophenanthridine alkaloids and protopine alkaloids	0.5 g/ewe/day	Adding into basal diet	Pelibuey ×Katahdin ewes	Female	20	NS	35 ± 2.3 kg	Gain efficiency and energy utilization efficiency (↑); scores for cellular dropsical degeneration, parakeratosis, and neutrophil infiltration of rumen epithelium (↓) [118].
Wheat bran feruloyl oligosaccharides	100, 200 mg/kg of feed	Mixing with basal diet	Dorper × Small-tail Han sheep	Male	50	2 months old	20.21 ± 3.36 kg	Improved ADG and FCR (↑), serum T-AOC levels, antioxidant enzymes activity, and glutathione content (↑); ruminal NH_3_-N content (↓) [119].
Red orange and lemon extract (flavanones, anthocyanins, hydroxycinnamic acids, ascorbic acid)	90 mg/kg of feed	Administration by gavage	Lambs	NS	120	4 days ± 12 h old	3.7 ± 0.2 kg	Activities of SOD, CAT, GPx (↑); MDA, 8-OHdG, IL-1β and IL-6 in plasma (↓) [120].
Lactobacillus fermented plant extracts with terrestrial plant extract (chicory, plantain, lucerne, leaf dock)	10 mL/ewe/d	Hung from the perimeter fence	Ewes	Female	60	94 ± 7 days old	29.6 ± 2.4 kg	Plasma TAS and GPx levels of ewes (ND); plasma TAS level (↑); GPx activity of their lambs (↓), indicating plant extracts increased maternal transmission of antioxidants to their offspring [121].
Fermented wheat bran polysaccharide	3 g/kg of diet	Mixing with basal diet	Crossed (Dorper × Mongolian sheep) lambs	Female	72	4 months old	34.82 ± 1.07 kg	ADG, apparent digestibility of DM, OM, and CP (↑), plasma levels of IgG, IgM, IL-10, and CAT activity (↑) [122].
Goats								
Polysaccharides-rich noni (Morinda citrifolia L.) fruit extract	0.4% of DM	Mixing with basal diet	Cashmere goats	Male	12	2 years old	45.44 ± 3.30 kg	Concentrations of TNF-α and IL-6, and the activities of T-SOD, TrxR, GPx, and catalase in serum (↑); levels of ROS and MDA in serum (↓); increased body weight of goats (↑) [112].
Astragalus Membranaceus	1% of DM	Mixing with basal diet	Cashmere goats	Female	24	12 months old	27.86 ± 1.61 kg	Myoglobin content, and the activities of some antioxidant enzymes (SOD and catalase) (↑), values of free radical scavenging ability and cupric reducing antioxidant capacity in goat muscle (↑); contents of metmyoglobin and MDA (↓) [115].
Nano-ammonium octamolybdate (nano-Mo) and *Macleaya cordata* extracts (MCE)	Nano-Mo (10 mg/kg); MCE (3000 mg/kg)	Mixing with basal diet	Nanjiang brown goats	NS	36	1 years old	40.9 ± 2.1 kg	Nano-Mo or combination of nano-Mo and MCE: Cu content in serum (↓); Fe and Mo contents in serum and liver of grazing goats (↑) exposed to contaminated grasslands with heavy metal;Nano-Mo, MCE, and combination of both: Hb, RBC, and PCV levels, and activities of serum SOD, GSH-Px, CAT and Cp (↑); blood WBC content and serum MDA content (↓) [123].
Oregano (*Lippia palmeri* Watts) extracts	In vitro: 100, 150 μg/mL; In vivo: 2.6% of DM	In vivo: mixing with basal diet	Anglo-Nubian goats	NS	12	NS	NS	In vitro: peripheral blood leukocytes content, nitric oxide production, phagocytosis, and SOD activities (↑); pro-inflammatory (TNF-α and IL-1β) transcription and antioxidant (CAT and GPX-4) genes (↓);In vivo: caproic acid production, and compounds related to the improvement of intestinal health (↑), which showed strong antioxidant capacity of oregano [124].

NS: not shown; (↑): increase; (↓): decrease; ND: no difference. Abbreviations: ADG, average daily gain; FCR, feed conversion ratio; IL-1β, interleukin 1β; TNF-α, tumor necrosis factor-alpha; GSH-Px, glutathione peroxidase; SOD, superoxide dismutase; T-AOC, total antioxidant capacity; CAT, catalase; 8-OHdG, 8-hydroxy- 2′-deoxyguanosine; IL-6, interlukin 6; GPx, glutathione peroxidase; TAS, total antioxidant status; T-SOD, total superoxide dismutase; TrxR, thioredoxin reductase; Hb, hemoglobin; RBC, erythrocyte count; PCV, packed cell volume; GSH-Px, glutathione peroxide; CAT, catalase; Cp, ceruloplasmin; GPX-4, glutathione peroxidase 4.
